# Comparing satisfaction with a participatory driven web-application and a standard website for patients with low back pain: a study protocol for a randomised controlled trial (part of the ADVIN Back Trial)

**DOI:** 10.1186/s13063-018-2795-0

**Published:** 2018-07-25

**Authors:** Allan Riis, Jan Hartvigsen, Michael Skovdal Rathleff, Tamana Afzali, Martin Bach Jensen

**Affiliations:** 1 Department of Clinical Medicine, Research Unit for General Practice in Aalborg, Aalborg University, Fyrkildevej 7, 1. Sal, 9220 Aalborg, Denmark; 2 Department of Sports Science and Clinical Biomechanics, Center for Muscle and Joint Health, University of Southern Denmark, Campusvej 55, 5230 OdenseM, Denmark; 3 Nordic Institute of Chiropractic and Clinical Biomechanics, Campusvej 55, 5230 Odense, Denmark

**Keywords:** Low back pain, Health information technology, Patient satisfaction, General practice, Participatory design, Advice, Patient education

## Abstract

**Background:**

Low back pain (LBP) is the most common musculoskeletal disorder and a leading cause of disability worldwide. It impacts daily life and work capacity and is the most common reason for consulting a general practitioner (GP). According to international guidelines, information, reassurance, and advice are key components in the management of people with LBP; however, the consultation time available in general practice for each patient is often limited. Therefore, new methods to support the delivery of information and advice are needed and online technologies provide new opportunities to extend the consultation beyond the GP’s office. However, it is not known whether GPs and people consulting their GP because of LBP will accept online technologies as part of the consultation. By involving patients in the development of online information, we may produce more user-friendly content and design, and improve patient acceptance and usage, optimising satisfaction and clinical outcomes. The purpose is to study satisfaction in people consulting their GP with LBP depending on whether they are randomised to receive supporting information through a new participant-driven web application or a standard reference website containing guideline-based information on LBP. It is hypothesised that patients offered information in a new web application will be more satisfied with the online information after 12 weeks compared to patients allocated to a standard website.

**Methods:**

Two hundred patients with LBP aged ≥ 18 years consulting Danish general practice will be randomly allocated 1:1 to either the new web application or standard online information in permuted blocks of two, four, and six. Patients with serious spinal diseases (cancer, fractures, spinal stenosis, spondyloarthritis), those without Danish reading skills or without online access, and pregnant women will not be included in the trial. Patient satisfaction measured by the Net Promotor Score after 12 weeks is the primary outcome. Patients will be aware of their allocation. GPs will be blinded unless informed by the patient. Assessors are blinded.

**Discussion:**

To our knowledge, this is the first trial evaluating whether involving LBP patients in the development of an online web application will result in higher patient satisfaction.

**Trial registration:**

ClinicalTrials.gov NCT03088774. Registered on 23 March 2017. Last updated on 14 March 2018.

**Electronic supplementary material:**

The online version of this article (10.1186/s13063-018-2795-0) contains supplementary material, which is available to authorized users.

## Background

Low back pain (LBP) is a leading cause of disability all over the world and affects people in all ages [1]. The point prevalence has been estimated to be 11.9% [2]. In the UK, direct healthcare costs for people with chronic LBP are twice as high when compared to people without LBP. Moreover, if indirect societal costs such as work absenteeism are included, the total costs are three times higher [3]. In Denmark, LBP is the most frequent reason for patients to consult general practitioners (GPs), resulting in 3.5 million annual consultations for a total population of 5.6 million [4]. The causes for LBP are often not known with certainty, but biological, psychological, and social factors all contribute to various degrees [5–7]. Most people who experience LBP are not severely affected, however many have ongoing pain or experience recurrence [6–8].

The GP is the primary contact healthcare professional and the gatekeeper to the more specialised part of the healthcare system in many countries including in Denmark. The GP is expected to triage the patient with LBP and rule out serious disease and underlying pathologies. After triage, the GP is expected to provide information on LBP, give reassurance, advise patients to stay active, inform patients on the use of analgesics, and consider supervised exercise therapy or manual therapy [9, 10]. Delivering this information can be time-consuming, many GPs are frustrated because they are unable to adhere to guideline recommendations due to the short consultation times [11]. A referral to supplementary primary care treatment with physiotherapists or chiropractors is one option, but such referrals are not feasible for all patients. Some patients may not want to consult another healthcare professional and others may not be willing or able to pay for supplementary care. Therefore, information technologies can potentially be used to extend the consultation and deliver evidence-based information and advice.

Patient information and advice can be provided to patients in a paper format [12], whereas information and advice delivered online can differentiate between several types of content (e.g. text, pictures, videos) and can be designed to match the target group [13]. Further, online information may be easily adjusted when and if guideline recommendations change and can be produced and maintained at low cost [14].

Online technology can improve accessibility and exchangeability of information, and thereby support self-management of LBP [15, 16]. Supporting patient self-care by use of web-based tailored interventions for other health conditions has been found effective [17]. However, the latest systematic review shows that evidence for effectiveness is lacking and it is difficult to conclude what will work for whom [18]. A personalised approach may address the individual biological, psychological, and social factors that are particularly important for the individual patient; however, individualisation of information and advice is resource demanding since healthcare professionals need to interact with both the technology and the patient. Digital web applications is one way of overcoming this by differentiating the information based on the patient’s bio-psycho-social profile through integration of information from valid and reliable questionnaires according to common characteristics [19, 20]. One such approach is the Stratified Targeted Treatment (STarT) Back Tool (SBT), previously found successful in subgrouping LBP patients in relation to prognosis [21]. Another approach to targeted advice can be the use of a pain monitoring model, aligning fluctuation of pain intensity in a patient with the advice given at different pain levels [22]. Furthermore, grading of activity is a third option to advise patients with LBP to be more active [23].

An important assumption behind the possible effects of web applications in healthcare is that patients actually use it. Therefore, patient preferences regarding delivery of health-related information and advice are important and should be identified, including preferred platforms for information, design, relevant content, and pitching the information correctly to improve comprehension and compliance. It is essential to know how patients with LBP – the end users – want to use technology to gather health-related knowledge. We therefore interviewed 15 patients with LBP who consulted their GP [24]. We found that the requirements for online material differed between patients, and domains such as design, customisation, usability, readability, and credibility all were important for patients’ satisfaction and acceptance of a homepage [unpublished material from our interview study]. These responses are in line with conclusions from a recent systematic review [25]. Among the different websites, the online Danish public portal for health ‘Patient Handbook’ [26, 27] was mentioned as the preferred site when searching for information and advice for LBP. The Patient Handbook is independent of industry, commercial-free and freely available on the official portal for the public Danish Healthcare Services. The portal is continuously updated and provides information about the Danish Healthcare Services and includes information about LBP and other health issues [27]. The editorial group consists of GPs as well as a broad range of other medical and allied healthcare specialists [27]. However, patients found the website difficult to navigate, hard to understand, and patients were insecure about whether different advice applied to them [unpublished material].

The aim of this study is to determine whether patients consulting general practice with LBP will be more satisfied with online information and advice after 12 weeks when using a new participant-driven web application compared to the electronic ‘Patient Handbook’.

## Methods

**Fig. 1:**
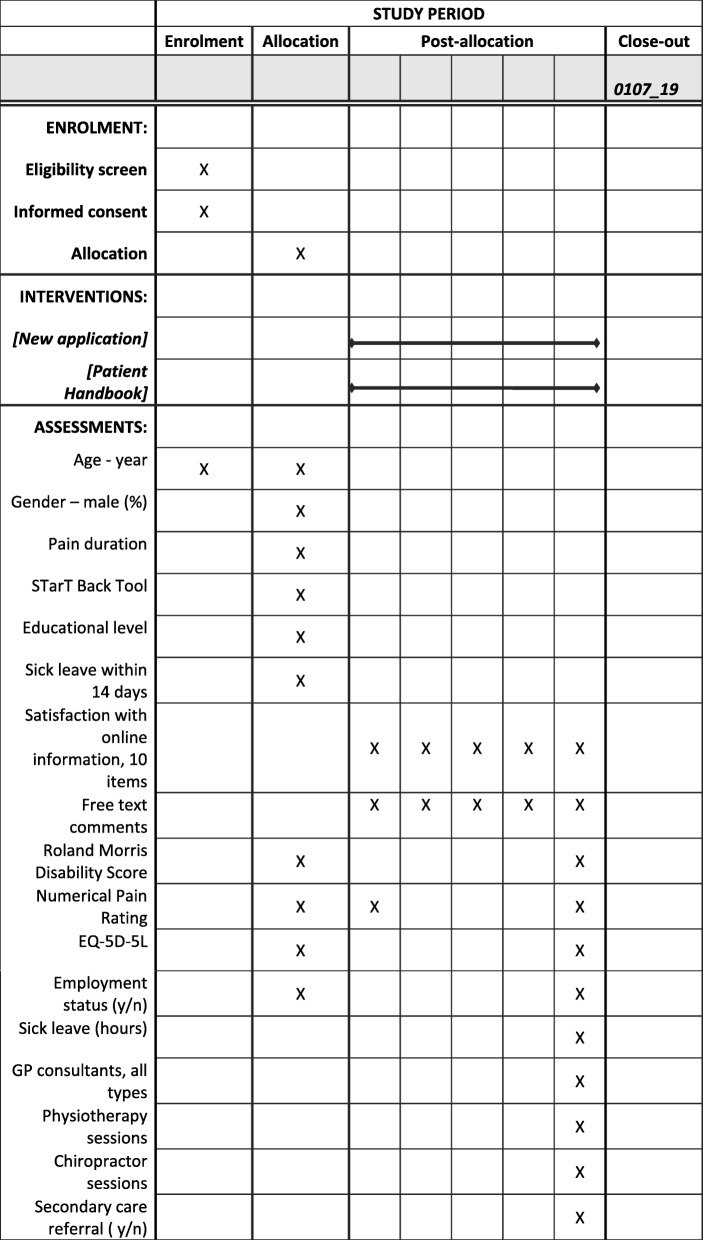
Timeline of the study. *NOTE:* Data from questionnaires integrated in the web application. Patients are asked to fill in questions following login at the given time point and before accessing the information on the homepage. In addition, number of entries at homepages will be collected

This is an assessor-blinded two-armed parallel group randomised controlled superiority trial allocating patients 1:1 to either the new web application or to the Patient Handbook [26]. The trial is planned accordingly to recommendations for interventional trials (SPIRIT) guidelines; see timeline in Fig. 1 and the SPIRIT checklist (Additional file 1). The trial is registered at ClinicalTrial.gov; NCT03088774 on March 23 2017.

### Recruitment and inclusion criteria

**Fig. 2:**
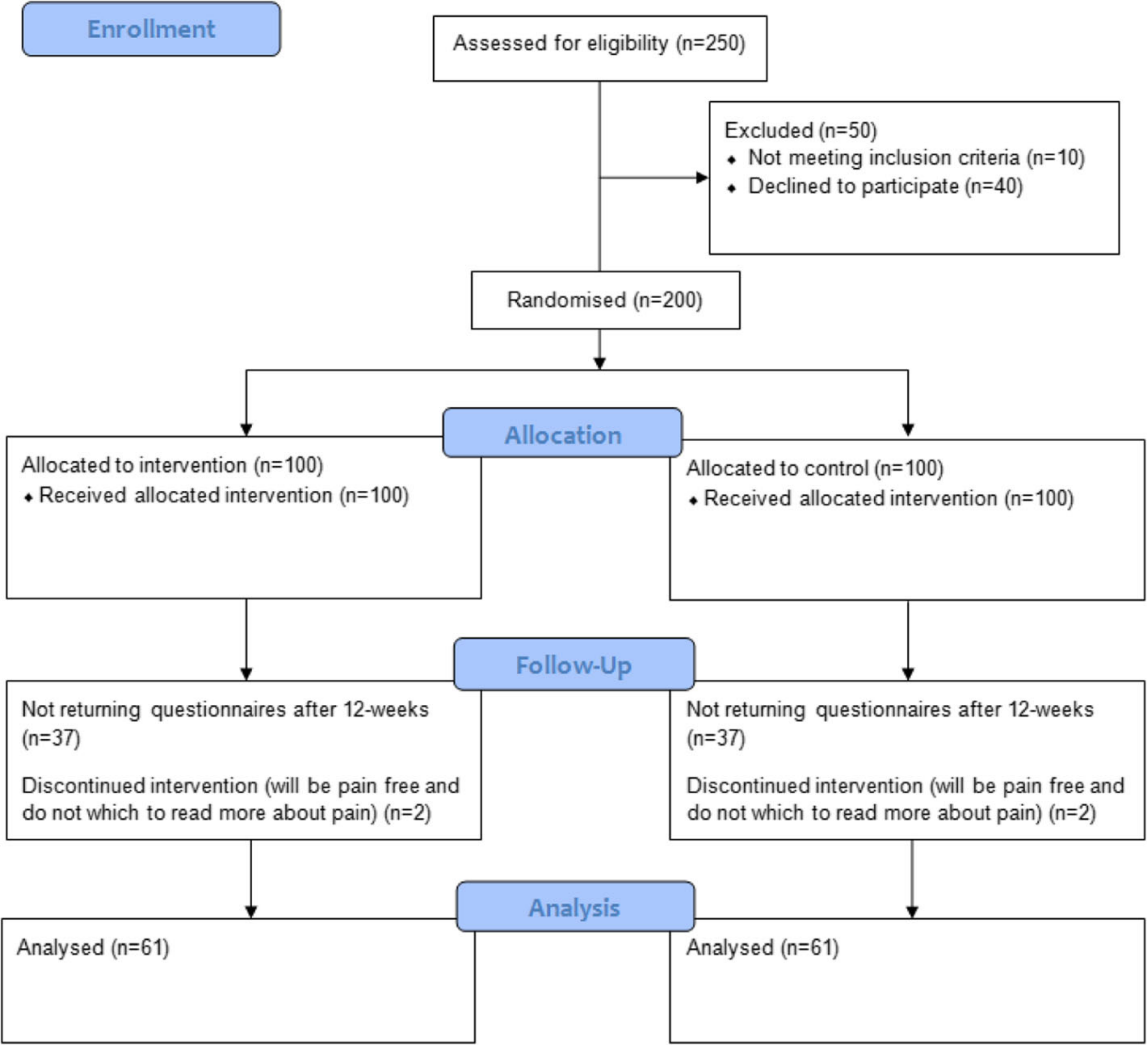
Flowchart

Ten Danish general practices will be included (five located in urban areas and five located in rural areas). The practices are expected to initially assess an average of 25 patients for eligibility and include 20 in each practice, resulting in a total sample of 200 patients (Fig. 2). Patients ≥ 18 years consulting with acute or chronic LBP with or without concomitant leg pain will be included regardless of pain level. Patients with spinal stenosis or serious underlying disease (e.g. signs of fracture, cauda equina syndrome, malignancy, osteoporosis, or spondyloarthritis), patients without Danish reading skills, patients without internet access, and pregnant women will be excluded. There will be an ongoing recruitment of practices and patients, and recruitment will be evaluated at weekly project meetings (AR and TA), where adaptive changes to meet barriers for recruitment will be discussed. The GPs will invite the patients to participate and provide the patients with study information and a link to formally sign up for the study.

### Interventions

**Fig. 3:**
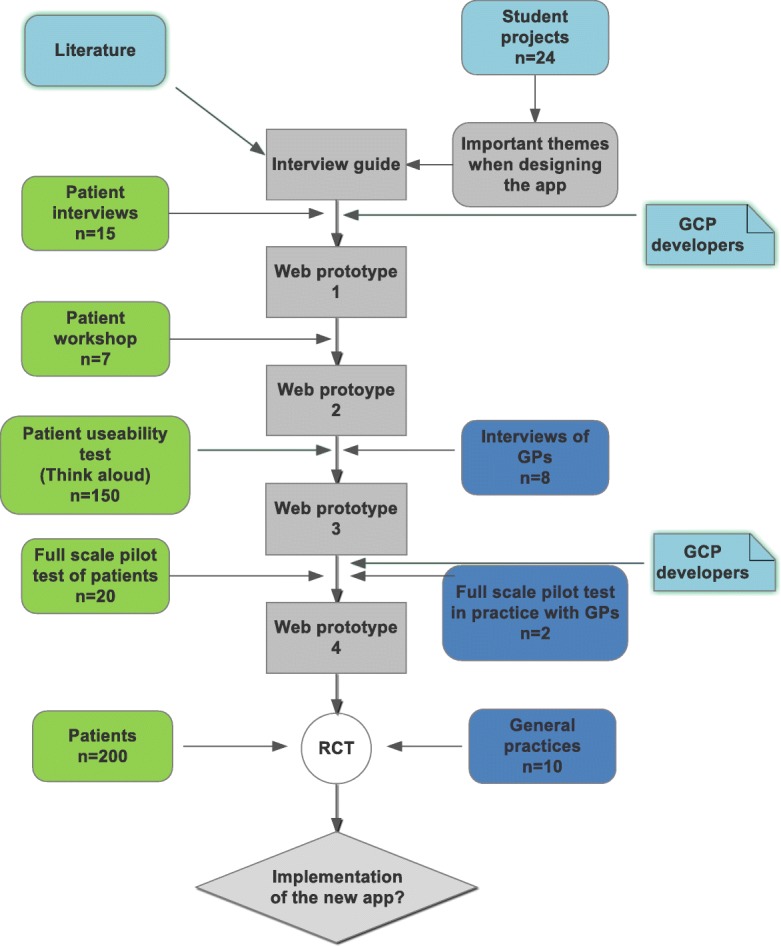
Participatory design of a new web application

The new online information material has a participatory design involving patients, GPs, and researchers (Fig. 3). Through two rounds of student projects (a total of 24 student groups of 1–5 bachelor students at Health Informatics at Aalborg University), we collected suggestions for important considerations when designing the web application. Based on a synthesis of their suggestions, we developed a semi-structured interview guide and conducted 15 interviews in patients’ homes [24]. We have then applied the patients’ preferences in the development of online information. Following the patient interviews, we invited seven respondents, of which two participated in the interviews, to participate in a workshop to discuss further adjustments to the web application. After this, the new web application was presented and discussed with eight GPs to ensure that it fits in their management of patients with LBP. Furthermore, we will test the web application on 50–200 people with LBP using the think-aloud while answering questions regarding readability, customisation, design, credibility, and usability of the web application, and we will adjust the web application (Fig. 3). Finally, new patients with LBP (*n* = 20) recruited from general practice will pilot test the web application including pilot testing the project setup. Two authors/guideline developers (MBJ and JH) have made corrections to ensure the delivery of guideline concordant information and advice. The online technology is developed together with Tempus Serva, Birkeroed and an IT consultant (Joachim Bøggild) at ProData, Viby, Denmark [28, 29]. The technology will be accessible to patients in the intervention group and available on smartphones, tablets, and computers (Additional file 2). Patients in the control group will be linked to information and advice on the Patient Handbook [26].

The interventions for the two groups are described accordingly to TIDieR [30] in Table 1. In both groups, patients will be able to access the information at any time. However, to support adherence in both groups, reminders will be emailed to all patients to re-access the online information after 1, 2, 4, 8, and 12 weeks. Furthermore, entries in both groups and the number of clicks and time spent on the web application in the intervention group will be used to monitor use during the 12-week period.

**Table 1:** Characteristics of the two interventions

	ARM 1: The intervention group	ARM 2: The control group
	The new online material	The Patient Handbook (standard website)
Participants	Adult patients consulting Danish general practice with low back pain	
Timing of intervention	Patients will be recruited consecutively. A patient will commence after a consultation at the GP and after providing informed consent online	
Intervention delivery mode	Interventions can be delivered on smartphones, tablets, or PCs	
Duration of intervention	Patients will receive reminders to access their interventions during the 12-week period, which corresponds to the total follow-up period	
Intervention provider	The new online information is developed by patients, GPs, and the research group	The Patient Handbook is developed by Sundhed.dk. Containing articles written by medical doctors and allied healthcare professionals
Intervention provider training	The provider/development group consists of an interdisciplinary team of researchers, with input from users (patients and GPs)	The provider group consists of a variety of healthcare professionals. Medical doctors are responsible for the content
Site of intervention delivery	The interventions will be delivered at patients’ homes, or at other places where patients choose to access the material	
Intervention process	Patients will be reminded to access the intervention after 1, 2, 4, 8, and 12 weeks. The interventions can be accessed as frequently as the patients prefer. Patients log on with username and password. Following login, patients will be led to a questionnaire containing two questions. At baseline and after 12 weeks the questionnaires are longer	
Intervention content	Guideline concordant information and advice for LBP produced by healthcare professionals an patients	Guideline concordant information and advice for LBP produced by healthcare professionals
Intervention materials	The interventions are delivered online. Some patients might choose to print exercise programmes or other materials on paper	
Fidelity of treatment	Fidelity will be measured by the number occasions entering the information (both groups) and number of clicks and duration spent on their online information material (intervention group)	

*GP* general practitioner, *LBP* low back pain

### Randomisation and blinding

At the first login, the patient will again be given study information and will be asked to provide consent online. Consenting patients will then be randomly allocated in blocks of two, four, and six, to the new web application or to the online ‘Patient Handbook’ (standard website) [26]. The allocation sequence will be delivered by statisticians at Aalborg University Hospital and integrated into the web application. All researchers will be blinded to group allocation. During the project, data will be stored on a server placed at Aalborg University. After completion of the last follow-up, MBJ will be unblinded and will prepare a data set with a dummy variable for allocation without free text information from patients and make this data set available for the assessors (AR and MSR) to analyse. AR, MSR, JH, and TA will be unblinded after presentation of the results of the primary and secondary outcomes to the research group. Patients will be aware of their allocation. GPs will only be aware of the allocation if the patient chooses to inform the GP.

### Primary outcome

Patients have not shown satisfaction with existing solutions for delivering online information, and if patients are not satisfied, they will probably refrain from seeking information and advice online [25]. What leads to satisfaction, however, varies among individuals and can be a challenge to measure. However, asking people “how likely is it that you would recommend this product to a friend or colleague?”, has been found related to patients’ satisfaction with a service and strongly correlated to future use of services [31]. The question is referred to as ‘The Ultimate Question’ [32]. The question has been widely applied for benchmarking in the industry, e.g. by Apple and LEGO [32]. Responses are provided on a numerical rating scale from 10 (extremely likely) to 0 (not at all likely). Responses from 10 to 9 are grouped as ‘promoters’, responses from 8 to 7 are grouped as ‘passively satisfied’, and responses from 6 to 0 are grouped as ‘detractors’ [31]. Each group are given a percentage, e.g., promotors (50%), passively satisfied (35%), and detractors (15%), by subtracting the percentage of detractors (15%) from the percentage of promoters (50%) you will get the net promoter score (35% in this example). The score has a maximum range from – 100 to 100 depending on the ability of a company to meet patients’ need but also depending on the services of the company. For further exploration of reasons for differences in net promotor score we will include detailed questions about satisfaction with design, customisation, usability, readability, credibility, and coping. The questions will be responded on an ordinal response rate (very, some, little, not at all). The questionnaire will be sent to the patients online and it will only be possible to tick off one box for each question. If the patient wants to change their decision while filling in the questionnaire, another box can be ticked off and the first choice will automatically be deleted. Patients will be asked about their satisfaction with online information after 1, 2, 4, 8, and 12 weeks. The proportion of patients being promotors after 12 weeks is the primary analysis for satisfaction. In addition, the proportion of patients with available data for The Ultimate Question will be interpreted as the completion rate, which will be reported for both groups.

### Secondary outcomes

Numeric pain rating (NPR) (0–10 points) [33], Roland Morris functional disability score (RMDQ, the Patrick version, 0–23 points) [34], EuroQol-5-dimension 5-level (EQ-5D-5 L) [35], employment status (y/n), sick leave (number of days during the study), number of contacts to general practice, number of contacts to physiotherapists, number of contacts to chiropractors, and contacts to secondary care (yes/no) are all measured after 12 weeks and are considered as secondary outcomes.

### Data collection and baseline variables

Patients will fill in a baseline questionnaire after their first login. The baseline questionnaire will include questions on age, gender, contact information, educational level, sick leave (number of hours during the last 14 days), employment status, pain duration, STarT Back Tool (SBT) questionnaire, NPR, RMDQ, and EQ-5D. After 1, 2, 4, and 8 weeks, patients will receive questions regarding their satisfaction with the online information material (0–10 points) and NPR. After 12 weeks, patients will be emailed a link containing questions addressing their satisfaction with the online information material, RMDQ, NPR, sick leave (number of days since study inclusion), employment status, EQ-5D, use of primary care healthcare services, referral to secondary care because of LBP, overall satisfaction with the combined treatment since study inclusion (0–10 points), and a free text box for any comments regarding the information material received. Figure 1 shows an overview of the data collection. Despite the amount of missing data between week 1 and week 8, all patients with missing data for the primary outcome after 12 weeks will be included in a reminding procedure. An automatic reminder will be generated at week 13 and week 14. If a reply is missing after 15 weeks, we will send a personal email and a postal letter with a questionnaire and a prepaid envelope, and after 16 weeks, we will call the patient. If the patient is reached by phone and still does not fill out the last questionnaire, we will ask patients to provide a reason for dropout.

### Early stopping rule and data monitoring

No harm to patients is expected. Consequently, the trial has no safety committee and the trial is not expected to be stopped prematurely.

### Sample size

During questionnaire development, we tested the Patient Handbook on 20 patients and found that 45% were promotors. We find it realistic that this proportion will be 75% in the web-application group. Therefore, we expect to find 45% promotors in the control group and 75% promotors in the intervention group. Based on a power of 90% and an alpha of 0.05, we will need to analyse 61 participants in each group. To allow for unequal group size and dropouts the study requires 200 patients.

### Statistical analysis plan

Analysis will follow the intention-to-treat principle and will be performed according to the CONSORT guidelines [36]. A statistical analysis plan will be made public available before collection of outcomes after 12 weeks. Baseline characteristics will be presented without significance testing between the groups as the mean and confidence intervals if the data is normally distributed, otherwise the median and range will be given. Binary variables will be presented as the count and proportions.

The proportion of patients being promotors after 12 weeks is the primary end point. In the case of missing data due to dropouts before week 12, the latest (at week 1 or later) will be carried forward. In case of no follow-up data for a patient, we will not impute missing values. The net promotor score will be presented in a secondary analysis as the difference between net promotor scores unadjusted for covariates without confidence interval and as a Fisher’s exact test including all scoring groups (promotors, passively satisfied, and detractors) together with a confidence interval.

NPR will be analysed for both groups (intervention or control) as a random effect in a linear mixed-effects model with allocation as the intercept in profile analyses with 12 weeks’ follow-up. The primary follow-up point is 12 weeks. NPR will be presented unadjusted and adjusted for factors which can be related to needs and healthcare utilisation (age, gender, and pain duration).

RMDQ, EQ-5D-5 L, employment status, sick leave, and number of contacts with primary care services are all considered secondary outcomes. These outcomes will be presented unadjusted and analysed with Fishers’ exact test, Student’s *t* test, or the Mann-Whitney test depending on the distribution of data. For binary outcomes, the reporting will include both absolute and relative effect sizes. We will conduct a cost-utility analysis comparing the intervention group with the control group from a healthcare perspective with a 12-week time horizon. All public paid primary care costs, such as public paid costs for GP contacts, physiotherapy services, and chiropractic services, will be included. Quality adjusted life years (QALYs) will be applied as a measure of effect based on EQ-5D-5 L. We will not include costs for developing and maintaining the new technology or other protocol-driven costs. Based on the relatively short time horizon, costs and effects will not be discounted.

Analyses will be performed using Stata 14.0 (Stata Corp, College Station, TX, USA), and 95% confidence intervals will be applied for all analyses.

### Dealing with missing values

Missing values caused by nonresponse was expected to appear in more than 5% of the study population. A list-wise deletion of cases was believed to remove a greater proportion of dissatisfied patients. Since the level of satisfaction was hypothesised to be different in the two groups, this was believed to cause a higher proportion of missing values in the control group and could cause bias by underestimating the true difference between the two groups. Consequently, imputation of missing values will be performed [37]. Missing values will be imputed for all time points. The imputation procedure will be based on the assumption of missing at random. The procedure will be conducted with the MI command in Stata where 50 data sets will be created. However, since the groups are exposed to two different interventions and the interventions are believed to influence satisfaction, the missing values will be replaced by multiple implementation with estimated values based on all available information, but the imputation model will be conducted separately for the two allocation groups. A more detailed description of the handling of missing values will be provided in a future Statistical Analysis Plan (SAP) publication.

### Data management

Data will automatically be saved on a secure server hosted by Aalborg University. Postal responses will be kept in a locked safe at the Research Unit for General Practice in Aalborg. Coding and cleaning of data will be performed by AR and supervised by MSR. Prior to the analysis and based on a dummy variable for the allocation, AR will present a SAP for MBJ, JH, TA, and MSR. When the analysis is accepted and published, AR and MSR will be unblinded.

## Discussion

Information and advice are recommended globally in the management of LBP. However, there are no online materials for LBP that have been developed together with patients.

A follow-up period of 12 weeks can be considered a limitation to this study. However, for the primary outcome, we believe 12 weeks is sufficient to study patient satisfaction. Despite good intentions to improve the care of patients with LBP, we cannot rule out that we might decrease the quality of care. In a previous British study involving patients in the choice of treatment modalities, the research group had to stop the trial after pilot testing because patients were worse off than when they were treated with usual care [38]. Therefore, a 12-week period is considered reasonable.

Even though patient satisfaction is often included in the battery of questions in LBP studies, it is rarely used as the primary outcome. We chose patient satisfaction as our primary outcome because our patient interviews revealed that satisfaction was considered essential for use of online information. Without satisfaction it is unlikely that patients will continue to use applications, which may in turn influence patients’ functional levels and pain levels.

This study will contribute with knowledge of the effect and cost-effectiveness of including patients in the development of a web application, which can support GPs in delivering advice and thereby extend the consultation provided by the general practitioner.

### Trial status

Recruitment will start July 2018 and is expected to continue until February 2019.

## Additional files

Additional file 1: SPIRIT checklist. *NOTE:* It is strongly recommended that this checklist be read in conjunction with the SPIRIT 2013 Explanation & Elaboration for important clarification on the items. Amendments to the protocol should be tracked and dated. The SPIRIT checklist is copyrighted by the SPIRIT Group under the Creative Commons “Attribution-NonCommercial-NoDerivs 3.0 Unported” licence. (DOCX 23 kb)

Additional file 2: Mock Ups. *NOTE:* Pictures from the homepage shown on PC and smartphone. (JPEG 60 kb)

## Abbreviations

EQ-5D-5 L: EuroQol 5-dimension 5-level
GP: General practitioner
LBP: Low back pain
NPR: Numerical pain rating
QALYs: Quality-adjusted life years
RMDQ: Roland Morris Disability Questionnaire
SAP: Statistical analysis plan
SBT: STarT Back Tool
