# Supplementary figures and images for: Comparing satisfaction with a participatory driven web-application and a standard website for patients with low back pain: a study protocol for a randomised controlled trial (part of the ADVIN Back Trial)
Source: Trials. 2018 Jul 25;19:399. doi: 10.1186/s13063-018-2795-0 (PMC6060464; doi:10.1186/s13063-018-2795-0)

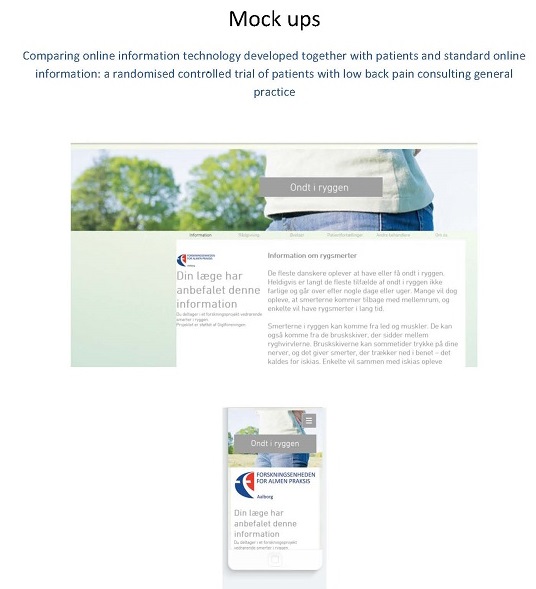

Supplement: Supplementary file 2 — Mock Ups. NOTE: Pictures from the homepage shown on PC and smartphone. (JPEG 60 kb) [file 13063_2018_2795_MOESM2_ESM.jpeg]
